# Suppression of *FUT1* attenuates cell proliferation in the HER2-overexpressing cancer cell line NCI-N87

**DOI:** 10.3892/or.2012.2120

**Published:** 2012-11-01

**Authors:** SADAYUKI KAWAI, SHUNSUKE KATO, HIROO IMAI, YOSHINARI OKADA, CHIKASHI ISHIOKA

**Affiliations:** Department of Clinical Oncology, Institute of Development, Aging and Cancer, Tohoku University, Aoba-ku, Sendai 980-8575, Japan

**Keywords:** α1,2-fucosyltransferase, Lewis Y antigen, HER2-overexpression, epidermal growth factor receptor, proliferation, short interfering RNA

## Abstract

Lewis Y (LeY) antigen is an oligosaccharide that is highly expressed at the cell surface in various human cancers. Increased LeY expression activates epidermal growth factor receptor (EGFR) and human epidermal growth factor receptor 2 (HER2) and promotes cell proliferation in EGFR-overexpressing cells. However, the effect of downregulation of LeY expression on cell proliferation in HER2-overexpressing cells remains unknown. *FUT1* encodes α1,2-fucosyltransferase, a key enzyme for LeY synthesis. We knocked down *FUT1* by short interfering RNA (siRNA) in four HER2-overexpressing human cancer cell lines, including NCI-N87, MKN7, SKBr3 and BT474. We investigated whether downregulation of LeY and alteration in the glycosylation status of these cells affect cell proliferation and HER2 activation. Knocking down *FUT1* expression markedly inhibited proliferation of NCI-N87, which highly expressed EGFR and was sensitive to EGFR deprivation. Furthermore, *FUT1* siRNA downregulated the total amount of HER2 protein, phosphorylation of HER2 and EGFR, and phosphorylation of extracellular signal-regulated kinase (ERK) in this cell line. Moreover, the marked downregulation of phosphorylation of HER2 and ERK was observed following short-time EGF-stimulation. These effects were not observed in the other three cell lines. Our results suggest that knockdown of *FUT1* downregulates HER2 signaling via EGFR downregulation. *FUT1* may serve as a new molecular target for HER2-overexpressing human cancers with activated EGFR signaling.

## Introduction

The cell membrane of mammalian cells comprises glycolipids, glycoproteins and proteoglycans; these carbohydrate structures undergo conformational changes during cellular differentiation and transformation (1). In particular, protein glycosylation accounts for the vast majority of post-translational processes that affect protein folding, stability, solubility and function (2). Glycosylation is catalyzed by glycosyltransferases. Among them, fucosyltransferases transfer an L-fucose sugar from a GDP-fucose donor substrate to an acceptor substrate (3). The fucosyltransferase gene family encodes enzymes that transfer fucose from α(1,2), α(1,3/4) and α(1,6) linkages to various glycans. *FUT1* and *FUT2* encode α(1,2)-fucosyltransferases, which transfer a terminal fucose residue from an α(1,2)-linkage to an existing galactose Type 1 or 2 precursor substance and form the H1 or H2 antigen as precursors of soluble ABH antigens, respectively (4). *FUT1* is ubiquitously expressed in the human body and preferentially expressed in erythroid tissues and vascular endothelial cells. *FUT2* is mainly expressed in the epithelial cells of the digestive and respiratory tracts (4). *FUT3*-*FUT7* and *FUT9* encode α(1,3)-fucosyltransferases, and their gene products transfer a fucose residue from an α(1,3)-linkage to galactose in H1 and H2 antigens to produce various Lewis antigens (5,6).

Antigens of the ABH and Lewis histo-blood group family can be found on the cell surface of various normal cells, mainly epithelial cells. However, the expression of various carbohydrate epitopes of this family is altered in carcinomas (7). For example, Lewis Y antigen (LeY), a Lewis antigen, is expressed in various cancer cells, including breast, ovarian and colorectal cancer; its expression is often associated with poor prognosis (8–12). In addition, forced *FUT1* and *FUT2* expression in human ovarian carcinoma-derived RMG-I cells increases activity of α(1,2)-fucosyltransferase and LeY antigen and promotes cell proliferation and resistance against anticancer drugs, such as 5-FU and carboplatin (13,14).

The molecular mechanisms through which overexpression of LeY antigen induces a malignant phenotype remain to be elucidated. However, increased LeY expression induced by *FUT1* and *FUT2* overexpression activates the epidermal growth factor receptor (EGFR) signaling and induces increase in mRNA expression and protein levels of human epidermal growth factor receptor 2 (HER2), a member of EGFR family (15). Furthermore, an *FUT1*- and *FUT2*-overexpressing cell line proliferates more aggressively than the parent cell line (15), suggesting that activation of EGFR and HER2 induces a malignant phenotype in human cancer cells.

HER2 is a transmembrane glycoprotein that is fucosylated by fucosyltransferase. It is involved in transmitting signals that stimulate cell division (16). HER2-overexpression is caused by amplification of the *HER2* gene and it is observed in various cancers (17,18), including breast and gastric cancer. Clinically, it is a molecular target of trastuzumab, a monoclonal antibody against HER2 (18,19). However, whether alteration of glycosylation affects cell proliferation in HER2-overexpressing cancer cells remains to be investigated. In this study, we examined the effect of *FUT1* suppression on the HER2 pathway and cell proliferation.

## Materials and methods

### Cell lines and cell culture

In this study, we used four HER2-overexpressing human cancer cell lines, NCI-N87 and MKN7 (derived from gastric cancer) and SKBr3 and BT474 (derived from breast cancer), and two cell lines, A431 and VMRC-LCD (derived from lung cancers), that do not overexpress HER2. NCI-N87, SKBr3, BT474 and VMRC-LCD were obtained from the American Type Culture Collection (Manassas, VA, USA). MKN7 and A431 were provided by the Cell Resource Center for Biomedical Research (Institute of Development, Aging and Cancer, Tohoku University, Sendai, Japan). All cell lines were maintained in RPMI-1640 medium (Sigma, St. Louis, MO, USA) supplemented with 10% heat-inactivated FBS (Gibco, Grand Island, NY, USA) and incubated at 37°C in a 5% CO_2_ humidified atmosphere.

### Preparation of siRNA and transfection

The following three pairs of siRNA oligomers were designed according to the sequence of human *FUT1* (GenBank accession number: NM_000148): *FUT1*-1 siRNA, 5′-AAAGGAUCUCUCAAGUC CGCGTT-3′ and 5′-CGCGGACUUGAGAGAUCCUUUTT-3′; *FUT1*-2 siRNA, 5′-GCUACACCGUGGAAAGACUTT-3′ and 5′-AGUCUUUCCACGGUGUAGCTT-3′; *FUT1*-3 siRNA, 5′-UCGAUGUUUUCUUUACACCAC-3′ and 5′-GGUGUAA AGAAAACAUCGACA-3′.

*FUT1-1* and *FUT1-2* siRNAs were designed based on a previous report (20) and the resource of Open Biosystems (http://www.openbiosystems.com), respectively. *FUT1-3* siRNA was designed using a web-based online software system (siDirect version 2.0, http://sidirect2.rnai.jp). These siRNAs were chemically synthesized by Hokkaido System Science, Co., Ltd. (Hokkaido, Japan).

Signal Silence EGF Receptor siRNA 1 and 2 (Santa Cruz Biotechnology, Inc., CA, USA) were used as EGFR-siRNA1 and 2, respectively. Negative control siRNA (Silencer Negative Control no. 1 siRNA) was obtained from Ambion, Inc. (Austin, TX, USA). Cells were seeded in a 6- or 96-well plate. After 24 h, the cells were transfected with siRNA (100 nM final concentration) using Dharmafect 2 reagent (Dharmacon, Lafayette, CO, USA).

### Measurement of mRNA expression by real-time PCR

Real-time polymerase chain reaction (PCR) was used to measure the mRNA expression of *FUT1* in the cells. Cells were plated at 1.5×10^4^ in a 96-well plate and transfected with siRNA (100 nM final concentration) as described above. Twenty-four hours post-transfection, the total-RNA from the cells was extracted using the RealTime ready cell lysis kit (Roche Diagnostics GmbH, Mannheim, Germany) and reverse transcribed using the Transcriptor First Strand cDNA synthesis kit (Roche Diagnostics GmbH) with oligo(dt) primer. Real-time PCR was performed using SsoFast™ EvaGreen Supermix (Bio-Rad, Richmond, CA, USA) and gene-specific primers in a thermal cycler CFX96 real-time PCR detection system (Bio-Rad). The primers used for amplification were *FUT1* F, 5′-AACGCCTCCTCTTCCTGTC-3′ and R, 5′-TGGGG TAGACAGTCCAGGTG-3′; glyceraldehyde 3-phosphate dehydrogenase (GAPDH) F, 5′-GAAGGTGAAGGTCG GAGTC-3′ and R, 5′-GAAGATGGTGATGGGATTTC-3′ (GenBank accession no. NM_002046). The designs of both primers have been previously described (21). Quantified data were normalized to GAPDH. The PCR program included 45 cycles of 95°C for 1 sec and 60°C for 5 sec. After the PCR reaction was completed, a melting curve analysis was performed. Each primer pair produced a single and sharp peak, thereby indicating that the primers amplified only one specific PCR product. No primer dimers were observed. All samples were amplified in triplicate.

### Western blotting

Cells were plated at 2.0×10^5^ in a 6-well plate and transfected with siRNA (100 nM final concentration) as described above. After 72 h, the cells were washed with cold PBS and harvested with lysis buffer [50 mM Tris-HCl (pH 8.0), 150 nM NaCl, 5 nM EDTA, 1% NP-40, protease inhibitor cocktail (Roche Diagnostics GmbH) and phosphatase inhibitor (Roche Diagnostics GmbH)]. In the short-time EGF stimulation experiment, 72 h post-transfection, the cells were starved in serum-free medium for 12 h and stimulated with EGF (10 ng/ml) for 10 min. The cells were washed immediately and harvested as described above.

Cell lysates were separated by 12.5% SDS-PAGE and blotted onto a PVDF membrane. The membranes were blocked with the Odyssey blocking buffer (Li-Cor Biosciences, Lincoln, NE, USA) and then probed with polyclonal anti-HER2 antibody (Dako, Carpinteria, CA, USA), monoclonal anti-phosphorylated HER2 antibody (Tyr1248; Santa Cruz Biotechnology, Inc.), monoclonal anti-EGFR antibody (Santa Cruz Biotechnology, Inc.), monoclonal anti-phosphorylated EGFR antibody (Tyr1068), monoclonal anti-GAPDH antibody (Santa Cruz Biotechnology, Inc.), anti-ERK1/2 antibody (Cell Signaling Technology, Inc.), anti-phosphorylated ERK1/2 antibody (Thr202/Tyr204; Cell Signaling Technology, Inc.) and anti-LeY antibody (Abcam, Cambridge, UK), followed by incubation with a goat anti-rabbit or a goat anti-mouse Alexa Fluor 680 IgG secondary antibody (Invitrogen, Carlsbad, CA, USA). Protein bands were detected and quantified using the Odyssey system (Li-Cor Biosciences).

### Cell proliferation assay

Cells were plated at 3.0×10^3^ in a 96-well plate and transfected with siRNA (100 nM final concentration) as described above. At 0, 72 and 120 h the cells were harvested and cell viability was determined using the Cell Counting kit-8 (Dojin Laboratories, Kumamoto, Japan), which measures mitochondrial succinate dehydrogenase activity. Briefly, 10 μl of 2-(2-methoxy-4-nitrophenyl)-3-(4-nitrophenyl)-5-(2,4-disulfophenyl)-2H-tetrazolium monosodium salt (WST-8) solution was added to each well. After a 2-h incubation at 37°C, the resulting water-soluble formazan dye was assayed by a microplate autoreader SpectraMax (Molecular Devices, Sunnyvale, CA, USA) at a wavelength of 450 nm with a reference of 630 nm. All experiments were performed in triplicate.

### Cell cycle analysis using fluorescence-activated cell sorter (FACS)

Cells were plated at 2.0×10^5^ in a 6-well plate and transfected with siRNA (100 nM final concentration) as described above. After a 72-h incubation, cells were treated with trypsin and fixed with 70% ethanol in PBS overnight. The cells were then washed once with PBS, incubated in the presence of RNase A (0.25 mg/ml) for 30 min at 37°C, collected by centrifugation at 200 × g for 5 min and stained with propidium iodide (50 μl/ml). The cells were filtered through a 50-μm pore size nylon mesh and analyzed for cell cycle using a FACS system (Beckman Coulter, Miami, FL, USA).

### Statistical analysis

All experiments were performed independently and in triplicate. Data are expressed as means ± standard error. Statistical data were analyzed using Student’s t-test. Significance was set at P<0.05.

## Results

### FUT1 knockdown by siRNA decreases LeY antigen expression

**Figure 1:**
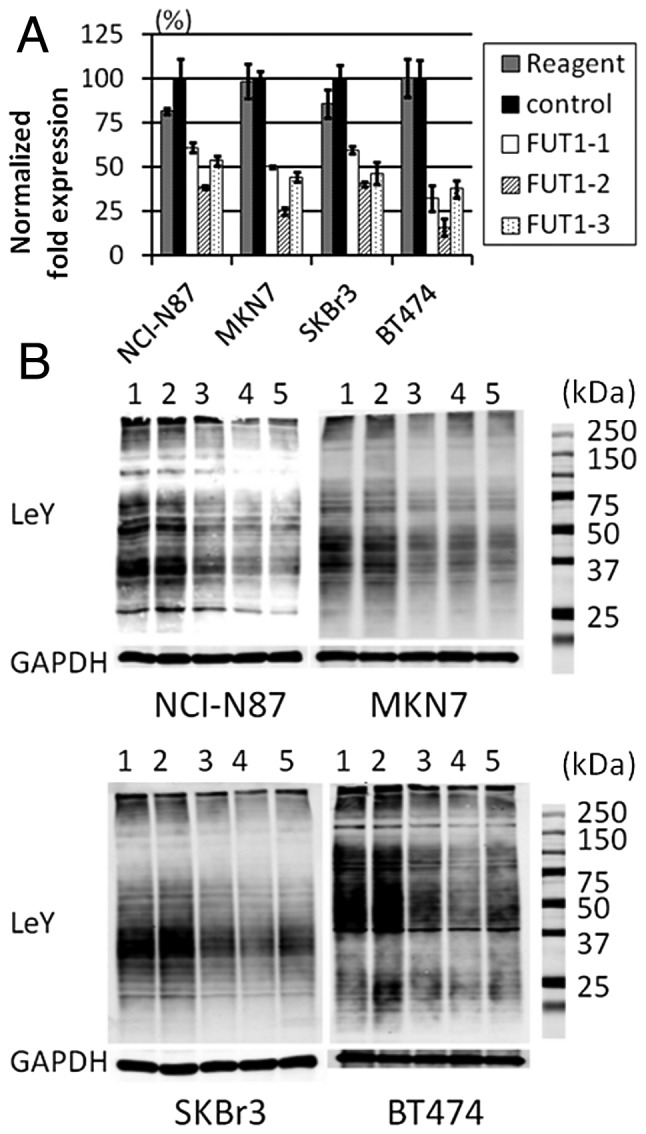
(A) Suppression of *FUT1* mRNA expression after transfection of siRNAs was analyzed by real-time PCR. The level of *FUT1* mRNA expression was determined and plotted as fold change relative to the control. Data were normalized to the amount of glyceraldehyde 3-phosphate dehydrogenase (GAPDH) mRNA. (B) Alteration of LeY expression after transfection of siRNAs in each whole cell lysate. GAPDH is shown as a loading control. Lane 1, reagent; lane 2, control; lane 3, *FUT1*-1; lane 4, *FUT1*-2; and lane 5, *FUT1*-3. A molecular weight standard is shown at the right side.

To detect the effects of *FUT1* siRNAs, we performed real-time PCR analysis (Fig. 1A) and western blotting of LeY antigen (Fig. 1B). *FUT1* mRNA and LeY antigen expression were reduced in all cells transfected with *FUT1* siRNAs, but not in those transfected with control siRNA. Compared with the cells transfected with control siRNA, the level of LeY expression in the cells transfected with *FUT1*-1, *FUT1*-2 or *FUT1*-3 siRNAs was *FUT1*-1 67.9, *FUT1*-2 44.5 and *FUT1*-3 46.9% in NCI-N87 cells; *FUT1*-1 50.5, *FUT1*-2 58.6 and *FUT1*-3 51.7% in MKN7 cells; *FUT1*-1 49.2, *FUT1*-2 33.3 and *FUT1*-3 62.3% in SKBr3 cells; and *FUT1*-1 54.1, *FUT1*-2 41.1 and *FUT1*-3 58.8% in BT474 cells. These results indicated that *FUT1* siRNAs efficiently reduced the levels of *FUT1* mRNA and LeY antigen expression.

### FUT1 knockdown inhibits cell proliferation in NCI-N87 cells, but not in other cell lines

**Figure 2:**
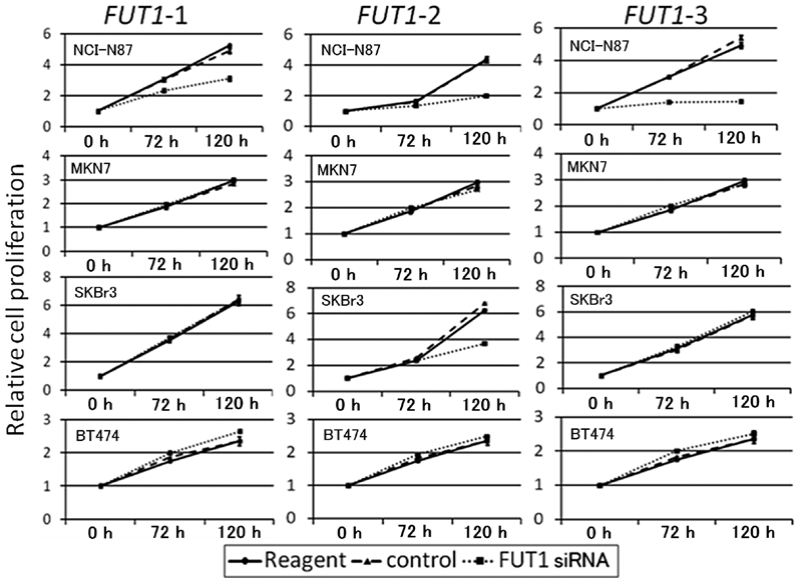
The growth curves after transfection with *FUT1* siRNA. Cells were assayed for growth 0, 72 and 120 h after transfection. The absorbance at 450 nm at 0 h was normalized to one. Absorbance at subsequent time points was plotted relative to the initial value.

To examine the effect of siRNA-mediated *FUT1* knockdown on cell growth, cell proliferation assays were performed for the four HER2-overexpressing cell lines. Data are shown in Fig. 2. *FUT1* siRNAs inhibited NCI-N87 cell proliferation 120 h post-transfection, whereas they did not inhibit proliferation in MKN7 or BT474 cells. Although *FUT1*-2 suppressed proliferation in SKBr3 cells, *FUT1*-1 and *FUT1*-3 did not. Therefore this was considered to be an off-target siRNA effect.

### FUT1 knockdown leads to apoptosis in NCI-N87 cells

**Figure 3:**
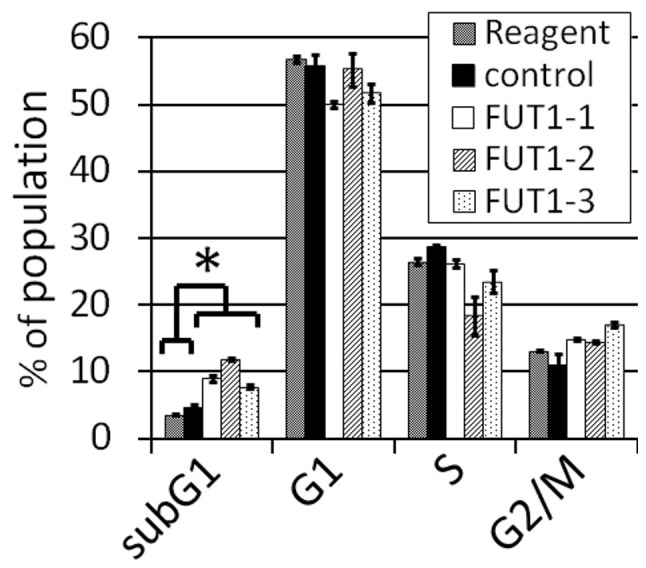
Analysis of the proportion of cells in each cell cycle phase after transfection with each *FUT1* siRNA in NCI-N87 cells. Values shown are average ± SE (n=3). Values of the sub-G1, G1, S and G2/M fractions are portions of the total population. ^*^P<0.01.

To examine whether *FUT1* knockdown changes the proportion of cells in each cell cycle phase, FACS analysis was performed for NCI-N87 cells (Fig. 3). All siRNAs significantly increased the subG1 fraction (P<0.01). The G2/M fraction also increased, but not significantly (P=0.09). These results indicate that the downregulation of *FUT1* mRNA and LeY antigen expression leads to apoptosis in NCI-N87 cells.

### FUT1 knockdown downregulates the expression of HER2 and the phosphorylated HER2 (pHER2) and phosphorylated ERK1/2 (pERK) in NCI-N87 cells

To elucidate the mechanism of cell growth inhibition induced by *FUT1* knockdown, HER2, pHER2, ERK1/2 and pERK were assessed by western blotting 72 h after transfection (we called it ‘normal cultural conditon’).

**Figure 4:**
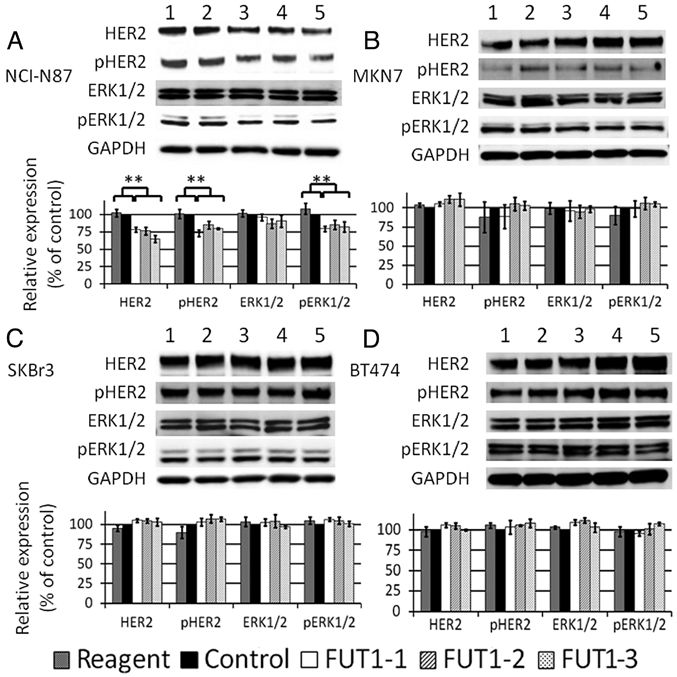
The effect of downregulating *FUT1* on protein expression and tyrosine phosphorylation of HER2 and ERK1/2 in normal culture condition. The bar chart shows the relative expression ratio of each protein by western blotting. Each value is calculated as the ratio of signal intensity compared to that of control. Data were normalized to signal intensity of glyceraldehyde 3-phosphate dehydrogenase (GAPDH). Lane 1, reagent; lane 2, control; lane 3, *FUT1*-1; lane 4, *FUT1*-2; and lane 5, *FUT1*-3. (A) NCI-N87, (B) MKN7, (C) SKBr3 and (D) BT474 cells. ^**^P<0.05.

Representative western blotting data and bar charts by triplicate experiments are shown in Fig. 4. *FUT1* knockdown significantly downregulated the total amount of HER2 and pHER2 in NCI-N87 cells (Fig. 4A). The amount of pERK also decreased, but the total amount of ERK remained unchanged. In contrast to NCI-N87, no significant changes were observed in MKN7, SKBr3 or BT474 cells (Fig. 4B-D).

### FUT1 knockdown strongly downregulates pHER2 and pERK following short-time EGF stimulation in NCI-N87 cells

**Figure 5:**
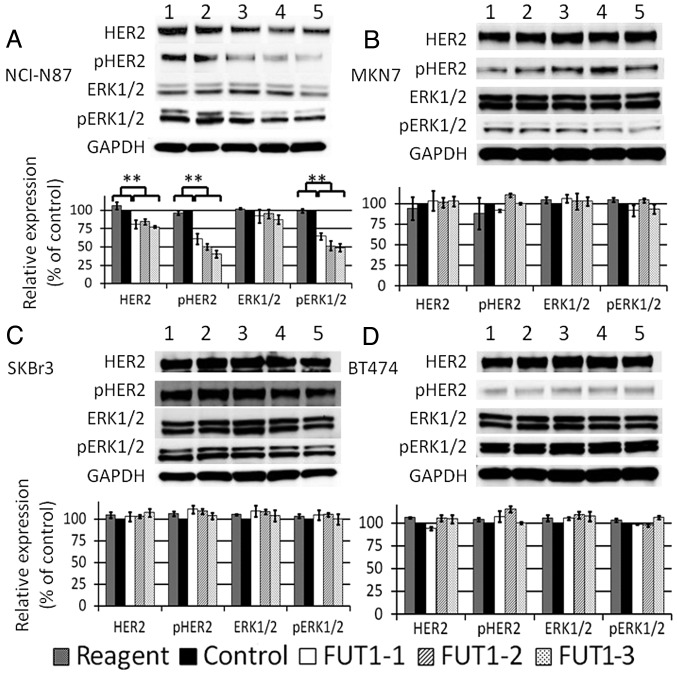
The effect of downregulating *FUT1* on protein expression and tyrosine phosphorylation of HER2 and ERK1/2 under EGF-stimulated condition. The bar chart shows the relative expression ratio of each protein found by western blotting. Each value is calculated as the ratio of signal intensity compared to that of control. Data were normalized to signal intensity of glyceraldehyde 3-phosphate dehydrogenase (GAPDH). Lane 1, reagent; lane 2, control; lane 3, *FUT1*-1; lane 4, *FUT1*-2; and lane 5, *FUT1*-3. (A) NCI-N87, (B) MKN7, (C) SKBr3 and (D) BT474 cells. ^**^P<0.05.

To examine whether short-time EGF-stimulation alters downregulation of HER2 and ERK1/2 by *FUT1* knockdown, we administered EGF for 10 min after starvation of the cells and assessed the amount of HER2, pHER2, ERK1/2 and pERK. The amount of pHER2 and pERK was markedly reduced in NCI-N87 cells (Fig. 5A). This reduction was more apparent than that of normal culture condition (Fig. 4A). Alterations in HER2 and ERK1/2 levels were similar to those observed in normal culture condition. In contrast, no significant changes were observed following EGF stimulation in MKN7, SKBr3 or BT474 cells (Fig. 5B-D).

### FUT1 knockdown downregulates EGFR signaling in NCI-N87

**Figure 6:**
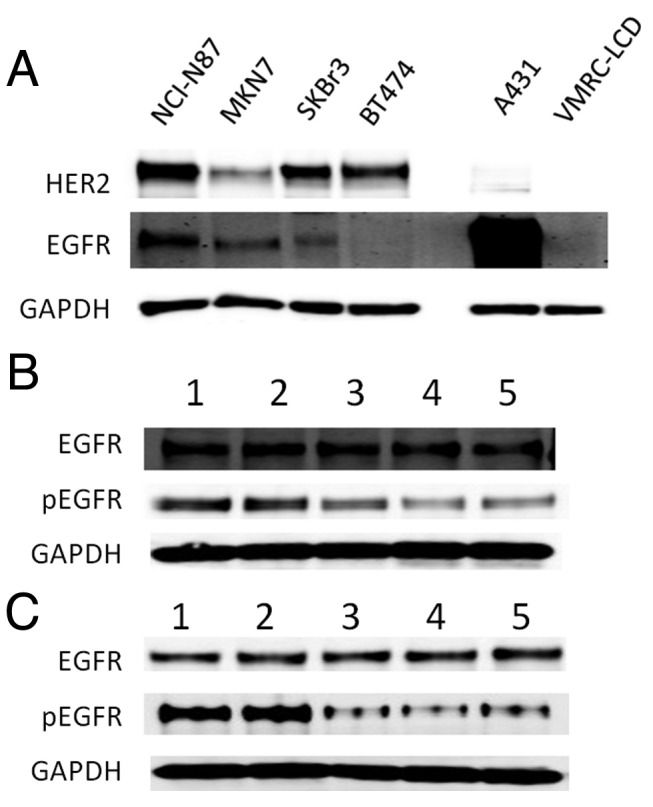
(A) HER2 and EGFR expression in each cell line was analyzed by western blotting. A431 is shown as a positive control for EGFR-overexpression and VMRC-LCD is shown as a negative control. (B) The effect of downregulating *FUT1* on protein expression and tyrosine phosphorylation of EGFR under normal cultural condition in NCI-N87 cells. Glyceraldehyde 3-phosphate dehydrogenase (GAPDH) is shown as the loading control. (C) The effect of downregulating *FUT1* on protein expression and tyrosine phosphorylation of EGFR under EGF-stimulated condition in NCI-N87 cells. Glyceraldehyde 3-phosphate dehydrogenase (GAPDH) is shown as the loading control. Lane 1, reagent; lane 2, control; lane 3, *FUT1*-1; lane 4, *FUT1*-2; and lane 5, *FUT1*-3.

To examine whether *FUT1* suppression affects EGFR signaling, first, western blotting for EGFR expression was performed (Fig. 6A). EGFR expression in each cell line was lower than that in the EGFR-overexpressing A431 cell line, whereas EGFR expression in NCI-N87 was higher than that in other HER2-overexpressing cell lines. Then, we investigated EGFR signaling of NCI-N87 after *FUT1* suppression. The result showed that phosphorylation of EGFR was downregulated in both normal cultural and EGF-stimulating conditions. The total amount of EGFR did not change in either of the conditions (Fig. 6B and C).

### Suppression of EGFR signaling downregulates HER2 signaling and proliferation of NCI-N87 cells

**Figure 7:**
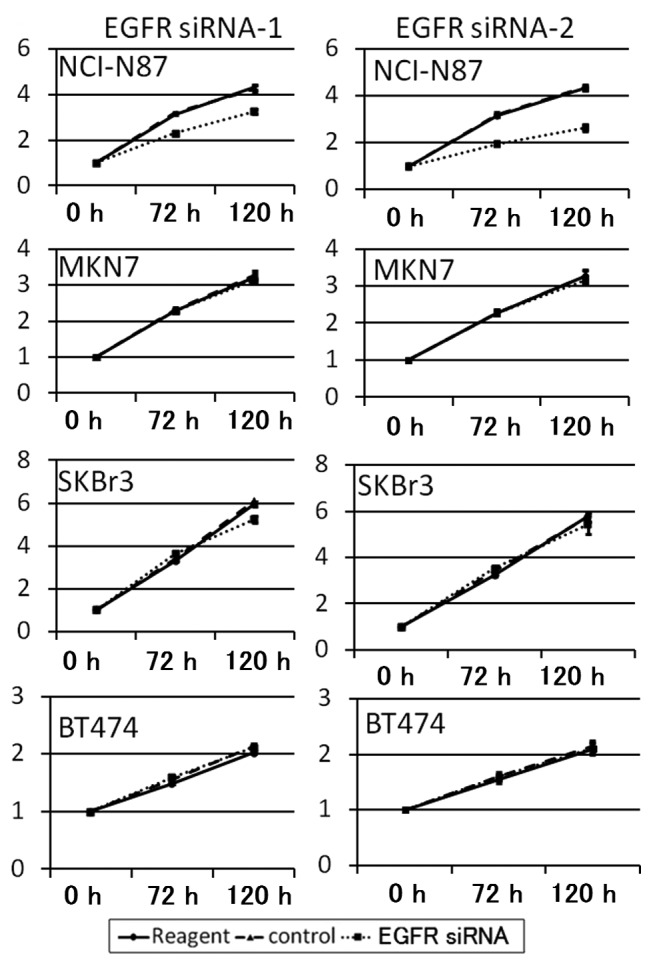
The growth curves after transfection with EGFR siRNA-1 and EGFR siRNA-2. Cells were assayed for growth 0, 72 and 120 h after transfection. The absorbance at 450 nm at 0 h was normalized to one. Absorbance at subsequent time points was plotted relative to the initial value.

**Figure 8:**
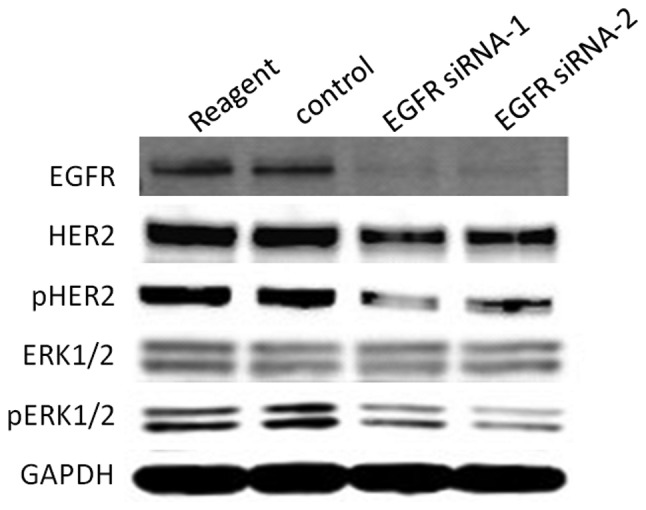
The effect of EGFR knockdown on protein expression and tyrosine phosphorylation of HER2 and ERK1/2 in NCI-N87 cells. Glyceraldehyde 3-phosphate dehydrogenase (GAPDH) is shown as the loading control.

To confirm the proliferation dependence of EGFR signaling the cell lines were transfected with EGFR siRNA. EGFR siRNA-1 and EGFR siRNA-2 suppressed the proliferation of NCI-N87 cells by 23.6 and 44.7%, respectively. However the proliferation of other cell lines were not changed by EGFR knockdown (Fig. 7). Next, to investigate whether EGFR suppression affects HER2 signaling in NCI-N87 cells, western blotting was performed. EGFR suppression downregulated HER2, pHER2 and pERK as well as *FUT1* suppression in NCI-N87 cells (Fig. 8). The results indicated that the proliferation of NCI-N87 cells was also dependent on EGFR signaling and EGFR suppression resulted in downregulation of HER2 signaling in this cell line.

## Discussion

LeY antigen belongs to the histo-blood group antigens and α1,2-fucosyltransferase is the key enzyme, which also *FUT1* and *FUT2* encode. Previous studies suggested that forced expression of α1,2-fucosyltransferase in RMG-I human ovarian cancer cell line caused overexpression of LeY antigen and promoted cell proliferation via activation of EGFR and HER2 (15). Furthermore, Palumberi *et al*(20) indicated that suppression of α1,2-fucosyltransferase inhibited the cell proliferation of the EGFR-overexpressing cell line A431. In the present study, we attempted to suppress *FUT1* gene by its specific siRNA and observe whether *FUT1* knockdown affected the cell proliferation of HER2-overexpressing cell lines.

Our results indicated that *FUT1* siRNA downregulated *FUT1* mRNA and altered fucosylation; it was shown by inhibition of LeY antigen expression, in four HER2-overexpressing cell lines. However, the effects on cell proliferation varied. In NCI-N87 cells, *FUT1* suppression decreased the total amount of HER2, pHER2 and pERK, and inhibited cell proliferation. However, *FUT1* suppression in MKN7, SKBr3 and BT474 did not alter HER2, pHER2 or pERK levels and did not affect cell proliferation.

In a previous study, HER2 inhibition led to suppression of cell proliferation in HER2-overexpressing cell lines (22–24). This observation is similar to our results and indicates that HER2 plays an important role in cell proliferation in HER2-overexpressing cells.

In addition, our study suggested that EGFR signaling was involved in *FUT1*-mediated inhibition of HER2 signaling in NCI-N87 cells. The experiment of EGFR siRNA transfection indicated that the proliferation of NCI-N87 cells was depentdent not only on HER2 signaling but also on EGFR signaling and EGFR suppression led to HER2 signaling inhibition. Previous studies indicated that cetuximab, a monoclonal antibody against EGFR, inhibits cell proliferation in NCI-N87 cells (25), but not in SKBr3 or BT474 cells (24). These results suggest that EGFR potently contributes to the proliferation of NCI-N87 cells.

We speculate that *FUT1* suppression leads to HER2 inhibition and cell proliferation via EGFR signaling inhibition through one or both mechanisms described below.

First, downregulation of EGFR by *FUT1* suppression may attenuate HER2 transcription. Liu *et al*(15) reported that *FUT1*-overexpression upregulated EGFR signaling and increased mRNA expression and protein levels of HER2. In our study, *FUT1* knockdown decreased the total amount of HER2 in NCI-N87 cells. Therefore, *FUT1* knockdown may have decreased HER2 levels by downregulating EGFR signaling. Since the level of attenuation of the total amount of HER2 was similar to that of pHER2, a reduction of the total amount of HER2 may cause downregulation of pHER2 and pERK in normal culture condition.

Second, *FUT1* suppression may attenuate EGFR and HER2 heterodimer formation. HER2 forms homodimers or heterodimers with other EGFR family proteins, undergoes autophosphorylation at specific tyrosine residues of its intracellular domain and mediates signal transduction (17). In addition, EGFR forms homodimers or heterodimers with other EGFR family proteins following ligand stimulation (25).

Following starvation and short-time EGF stimulation, phosphorylation of HER2 and ERK1/2 was markedly reduced in *FUT1*-suppressed NCI-N87 cells. Zhang *et al*(26) reported that the suppression of *FUT1* and FUT4 reduced LeY antigen, decreased binding of EGF to EGFR and resulted in inhibition of cell proliferation. In addition, some reports have shown that fucosylation on EGFR alters the binding affinity of EGF to EGFR and affects EGFR dimerization (2,27,28).

Hence, we propose that *FUT1* suppression caused an alteration of fucosylation and attenuated EGF-mediated EGFR and HER2 heterodimerization.

Besides, our results indicated that apoptosis occurred in *FUT1*-mediated growth inhibition in NCI-N87 cells. G2/M fraction also tended to increase but not significantly. Previous studies revealed that HER2 inhibition by trastuzumab caused apoptosis in some HER2-overexpressing cell lines, e.g. SKBr3 or Calu-3 (29). However, it did not cause apoptosis in SKOV-3 which had HER2-overexpression (30). Hence, it is possible that HER2 suppression causes various effects on cell proliferation among each cell line.

Lapatinib is a dual tyrosine kinase inhibitor for EGFR and HER2 and is used to treat trastuzumab-resistant HER2 positive cancers. Redundant signaling from other EGFR family members is one of the molecular mechanisms of drug resistance to trastuzumab (31). Inhibition of EGFR and HER2 signaling is one strategy for treating trastuzumab-resistant HER2 positive cancers.

The role of fucosylation in cell proliferation is not completely understood. However, our results demonstrate that *FUT1* knockdown results in the inhibition of cell proliferation and reduction of HER2, pHER2 and pERK in NCI-N87 cells. The reduction of pHER2 and pERK seems to depend on the reduction of EGFR signaling caused by inhibition of fucosylation. Further studies are necessary to identify a biomarker to predict which HER2-positive cancer cells are sensitive to *FUT1* inhibition. The development of a fucosyltransferase inhibitor may constitute a novel drug for trastuzumab-resistant HER2 positive cancers.
